# Combinatorial assessment of ctDNA release and mutational burden predicts anti‐PD(L)1 therapy outcome in nonsmall‐cell lung cancer

**DOI:** 10.1002/ctm2.8

**Published:** 2020-05-06

**Authors:** Wenfeng Fang, Yuxiang Ma, Jiani C. Yin, Huaqiang Zhou, Fufeng Wang, Hua Bao, Ao Wang, Xue Wu, Shaodong Hong, Yunpeng Yang, Yan Huang, Hongyun Zhao, Yang W. Shao, Li Zhang

**Affiliations:** ^1^ State Key Laboratory of Oncology in South China, Collaborative Innovation Center for Cancer Medicine Sun Yat‐sen University Cancer Center Guangzhou China; ^2^ Nanjing Geneseeq Technology Inc. Nanjing China; ^3^ Department of Medical Geneseeq Technology Inc. Toronto Ontario Canada; ^4^ School of Public Health Nanjing Medical University Nanjing China

Immune checkpoint blockade has revolutionized the treatment landscape in a multitude of advanced cancers.^1^ However, only a small subset (∼20%) of unselected patients derived durable clinical benefit (DCB).^2^, ^3^ As a consequence, substantial efforts have been made to improve immunotherapy outcome, including approaches to combine immunotherapy with other anticancer treatments^4^, ^5^ and identification of biomarkers that could enrich for potential responders.^6^, ^7^, ^8^, ^9^ In patients with advanced nonsmall‐cell lung cancer (NSCLC), a number of biomarkers that can predict clinical benefit from anti‐PD‐(L)1 monotherapies have been identified,^2^, ^6^, ^8^, ^10^, ^11^, ^12^, ^13^, ^14^, ^15^, ^16^ including high tumor mutational burden (TMB), which reflects the overall neoantigen load.^13^ However, most current studies are based on tissue samples, which can be inadequate for multiple testing in patients with advanced disease. Liquid biopsy utilizing circulating tumor DNA (ctDNA) provides an alternative and minimally invasive source for molecular testing in the absence of tissue samples. Plasma‐based next‐generation sequencing (NGS) testing has demonstrated its potential for complementing tissue testing in the identification of actionable mutations and resistance mechanisms. In contrast to tissue samples, plasma has its unique advantages in long‐term disease monitoring and overcoming tumor heterogeneity. Although blood TMB (bTMB) is a promising and clinically accessible biomarker, few studies exist evaluating its predictive value.^17^, ^18^, ^19^, ^20^ Furthermore, TMB alone is insufficient to completely enrich for potential responders, and therefore, additional predictive biomarkers and new approaches that optimize response prediction are urgently needed.

Previously, our group demonstrated that tissue TMB (tTMB) estimated by a 422‐cancer‐gene panel (GeneseeqPrime) in NSCLC patients is associated with clinical benefit from anti‐PD‐(L)1 therapies.^15^ In the current study, we performed targeted NGS of plasma ctDNA from a cohort of 97 patients, and analyzed the correlation between bTMB and clinical outcome. We evaluated the concordance between tTMB and bTMB using matched samples, as well as its impact on the enrichment of responders. Using data from two large randomized trials, we also explored the predictive potential of ctDNA release in NSCLC treated with PD‐(L)1 blockade.

A total of 108 patients with NSCLC were treated with anti‐PD‐(L)1 monotherapy agents at Sun Yat‐sen University Cancer Center between December 2015 and August 2017, data cutoff in January 2019. According to our eligibility criteria, which were the same as previously specified,^15^ a total of 97 NSCLC patients with evaluable radiological results, sufficient clinical information and baseline blood samples were analyzed (Figure S1). The median follow‐up time was 637 days. DCB was defined as the percentage of patients who responded or had stable disease (SD) lasted >6 months; nondurable clinical benefit was defined as the percentage of patients who experienced PD or less than 6 months of SD. Table S1 summarized the baseline clinical characteristics of the cohort. Plasma from 97 patients, as well as matched tissue samples from 66 patients, were profiled using targeted NGS with a 422‐cancer‐relevant gene panel (GeneseeqPrime)^15^ in the CLIA‐ and CAP‐accredited Geneseeq laboratory (Nanjing, China). The mean coverage depth was 143× for controls, 1341× for tissues, and 4185× for cfDNA samples. TMB estimation was performed using clinically validated procedures as previously described.^15^

Analysis of the entire cohort revealed inadequate performance of bTMB in predicting patient outcome. While increasing cut‐points of bTMB showed a clear monotonic relationship with progression‐free survival (PFS) outcome, significant PFS difference was found between bTMB‐high and ‐low patients only at cut‐points of 13 and above (Figure S2). In this regard, bTMB assessment identified less than 20% of patients who may derive PFS benefit, showing no enrichment of responders compared with the unselected population.

We have previously established the predictive value of tissue‐based TMB in our cohort^15^ and reasoned that the failure of bTMB to predict PFS benefit must owe to the technical aspect(s) of ctDNA detection in liquid biopsy. Thus, we evaluated the influence of cfDNA concentration and ctDNA maximum somatic allele frequency (MSAF) on bTMB assessment (Figure S3). cfDNA concentration displayed a weak positive correlation with ctDNA MSAF (Spearman ρ = 0.28, *P* = .007) and no significant correlation with bTMB (Spearman ρ = 0.11). On the other hand, a strong correlation between ctDNA MSAF and bTMB was observed (Spearman ρ = 0.76, *P* < .001). Consistent with previous studies,^17^ we further showed that a low ctDNA MSAF negatively impacts tissue‐plasma TMB concordance (Figure S4A‐C). At ctDNA MSAF cut‐points of 2% and above, the correlation between bTMB and tTMB became sufficiently high (Spearman ρ > 0.7), and thus samples with ctDNA MSAF <2% were excluded (Figure S1). Increasing cut‐points of bTMB again demonstrated a clear monotonic relationship with improved PFS outcome (Figure 1A). At bTMB cut‐points of 11 and above, bTMB‐high patients experienced significant PFS benefit compared with bTMB‐low patients (Figure 1A). Unlike the MSAF‐unadjusted cohort, the prevalence of bTMB‐high (≥11) was 32% in patients with MSAF ≥2%. High bTMB was associated with greater DCB (34.8 vs 22.5%, *P* = .39; Figure S2B) and significantly prolonged PFS (mPFS, 110 vs 60 days, hazard ratio [HR] = 0.54 [95%CI, 0.31–0.92]; Figure 1B).

**FIGURE 1:**
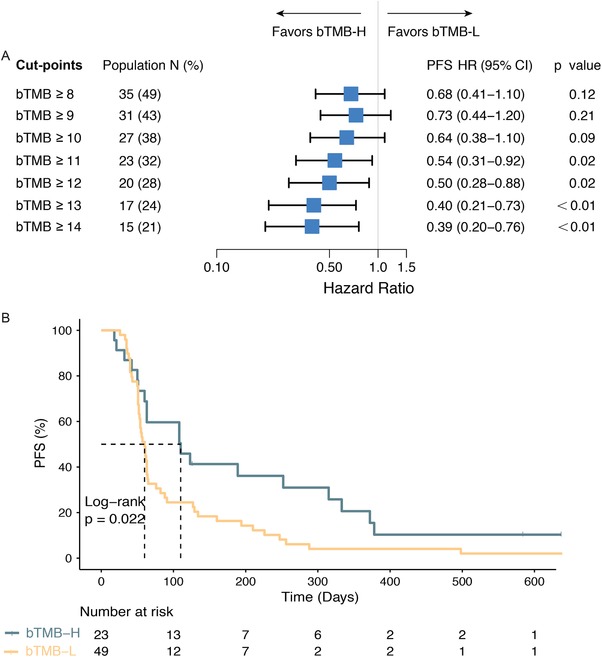
Panel‐assessed bTMB is associated with clinical benefit following MSAF adjustment. A, Forest plot of HRs for PFS comparing patients (ctDNA MSAF≥2%) at varying cut‐points of bTMB. Note that at bTMB ≥ 11, significant PFS differences were observed between bTMB‐high and bTMB‐low patients. B, Kaplan‐Meier estimates comparing PFS in patients with bTMB‐H (bTMB ≥ 11) and bTMB‐L (bTMB < 11; Log‐rank *P* = .022).

Despite substantial tumor heterogeneity in NSCLC, bTMB demonstrated a strong correlation with tTMB (Spearman ρ = 0.71; Figure 2A). To rule out the possibilities of sampling biases associated with a single tumor biopsy or net output of ctDNA that does not truly represent the mutational load of the dominant tumor clones, we focused on paired samples that were deemed TMB‐high by both tissue and blood assessments. Interestingly, such patients derived a greatly improved DCB benefit (42.9 vs 22.2%, *P* = 0.17; Figure 2B and Figure S3A) and PFS outcome compared with patients with low TMB as assessed using either sample type (mPFS, 156 vs 59 days, HR = 0.47 [95%CI, 0.24–0.91]; Figure 2C and Figure S3B). As plasma testing may be skewed by the net output of ctDNA from diverse subpopulations of cancer cells, it is not surprising that while tTMB or bTMB assessment alone is able to identify responders to immunotherapy, concordant bTMB and tTMB status has a greatly refined predictive potential.

**FIGURE 2:**
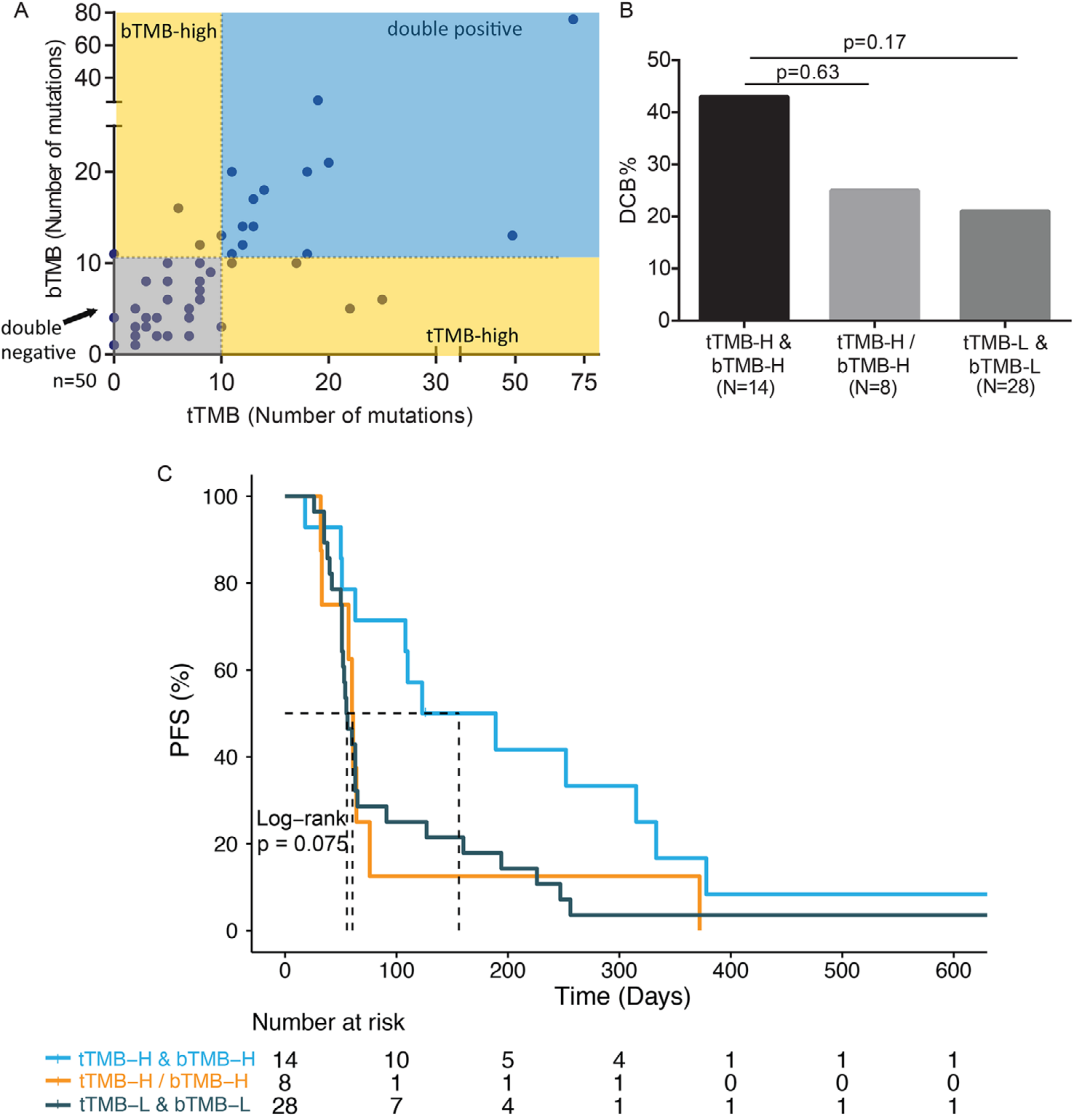
Concordance between bTMB and tissue TMB (tTMB) guides more precise prediction of clinical outcome. A, Correlation between bTMB (ctDNA maximum somatic allele frequency, MSAF, ≥2%) and tTMB (Spearman ρ = 0.71). B and C, Comparisons of (B) durable clinical benefit (DCB) rates and (C) PFS in matched tissue and plasma samples among patients with concordantly high (tTMB‐H & bTMB‐H), low (tTMB‐L & bTMB‐L), or discordant (tTMB‐H/bTMB‐H) bTMB and tTMB status.

Although a strong correlation between tTMB and bTMB was achieved by excluding patients with low ctDNA release, whether such patients may derive benefit from PD‐(l)1 blockade is unknown. Indeed, ctDNA release reflects tumor anatomic and metabolic burden,^22^ and may correlate with patient outcome. Thus, to further enrich for potential responders, we analyzed patients according to their MSAF status. As expected, of the 25 patients with low MSAF, eight (32.0%) patients experienced DCB, with an mPFS of 129 days, which was longer than those with high MSAF (Figure S4A). Notably, all of the three “low‐releasers” with discordant bTMB‐tTMB had low bTMB and high tTMB and experienced DCB and long PFS of 300 days and over. Thus, insufficient ctDNA release not only masked the predictive value of bTMB, but also reflected the impact of tumor metabolic load on patient outcome. To account for the influence of MSAF on patient outcome and bTMB, we performed combined analysis of MSAF and bTMB on the entire study cohort. Remarkably, our results revealed that patients with low ctDNA release experienced similar PFS benefit as those with high bTMB from PD‐(L)1 blockade (Figure 3A). Importantly, this finding was externally validated using data from two large randomized trials, POPLAR and OAK (Figure 3B and Figure S4B and C). As a consequence, our analysis led to an enrichment of potential responders from 24% to at least 50% following the integration of MSAF and bTMB into the process of patient selection for immunotherapy (Figure 3C).

**FIGURE 3:**
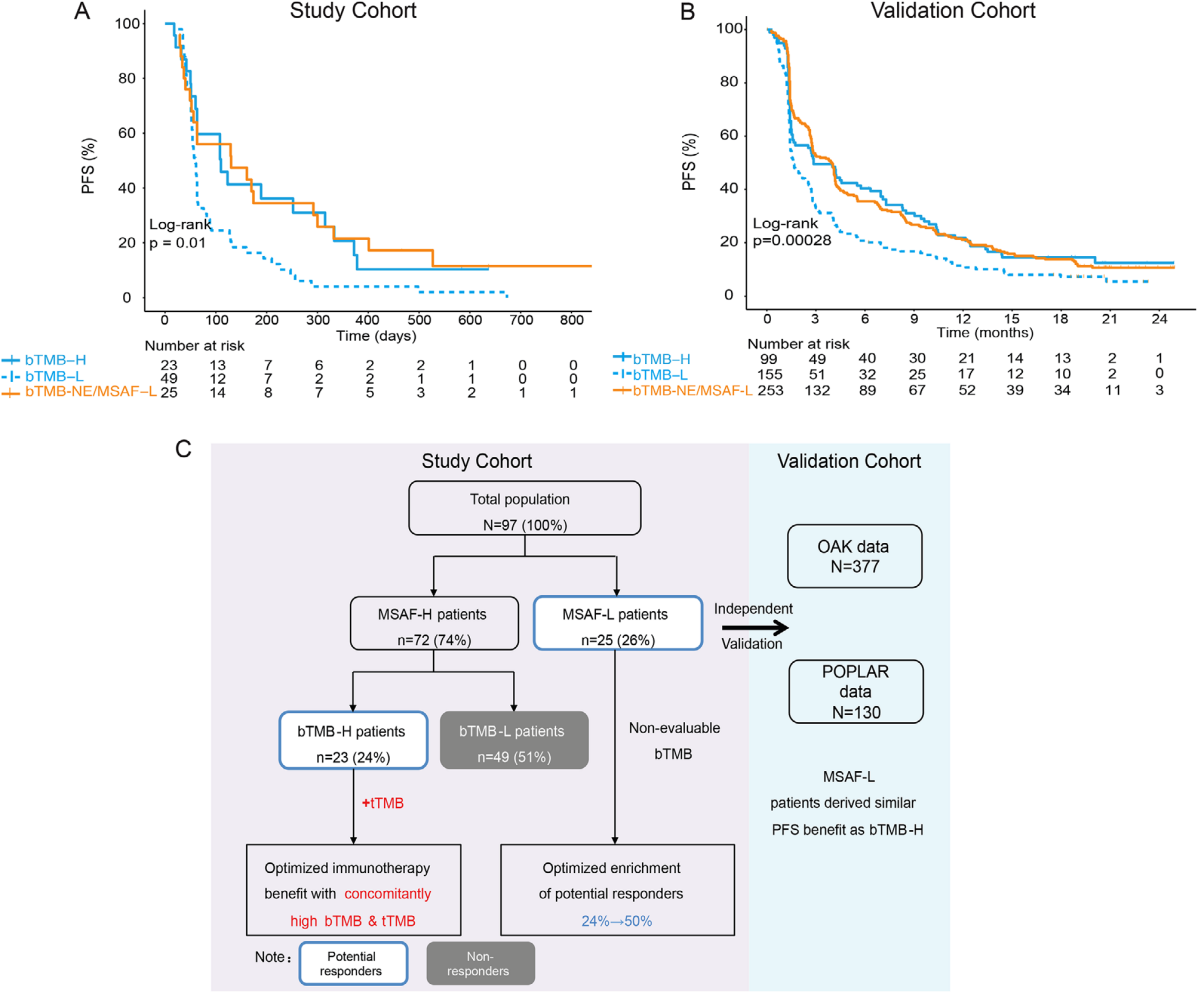
Low ctDNA MSAF allows for enrichment of responders to immunotherapy. A and B, Kaplan‐Meier estimates of PFS in the (A) full analysis set; (B) combined analysis set of the POPLAR and OAK studies comparing patients of different bTMB and ctDNA MSAF status. Note that MSAF‐low patients, which accounts for a substantial population of patients, derived similar PFS benefit as the bTMB‐high population. bTMB‐NE, non‐evaluable bTMB. C, Graphical summary of the overall study results. In patients with sufficient ctDNA release (MSAF‐H), bTMB with concordant tTMB status is associated with a refined predictive power for PFS outcome from anti‐PD‐(L)1 monotherapy. On the other hand, in those with low ctDNA release and, consequently, non‐evaluable bTMB, significant PFS benefit can be derived, with an overall enrichment of over 50% of patients who potentially respond. Independent validation using the OAK and POPLAR datasets confirmed ctDNA MSAF as a biomarker for response to immunotherapy.

There are two potential mechanisms by which low ctDNA release influences outcome prediction by bTMB. Low ctDNA MSAF, and resulting “low bTMB,” oftentimes does not truly reflect the tumor's mutational status. Indeed, we find that over 18% of “low releasers” have low bTMB but high tTMB. On the other hand, ctDNA release is associated with overall tumor load and metabolic burden, and in many disease settings, correlates with patient outcome and response to therapies. Consistent with this notion, we find that patients with low ctDNA MSAF obtained PFS benefit from anti‐PD‐(L)1 therapies. Thus, rather than excluding the “low releasers” on the basis of inaccurate representation of bTMB, incorporation of MSAF status enriches for a large subset of potential responders. Given the association between ctDNA levels and prognosis in a number of cancer types,^23^, ^24^ it is conceivable that ctDNA MSAF might also be a prognostic biomarker. However, further investigations are necessary as our study design precluded the evaluation of the prognostic value of ctDNA MSAF or bTMB.

In conclusion, our study validated the clinical utility of panel‐estimated bTMB as a biomarker to identify NSCLC patients who may respond to immunotherapy. We also provided the first clinical evidence showing that matched tissue and plasma testing should be recommended when selecting patients for immunotherapy, as bTMB‐tTMB discordance negatively impacts prediction of clinical outcome. On the other hand, we identified ctDNA MSAF as a novel biomarker for predicting immunotherapy outcome. Importantly, low ctDNA release is associated with significant PFS benefit and integrated analysis of MSAF, and bTMB allows for a greatly enhanced enrichment of potential responders (Figure 3C). Our results have important clinical relevance and significance in the detection of blood‐based ctDNA as a source of molecular biomarkers for cancer immunotherapy.

## CONFLICT OF INTEREST

The authors declare that there is no conflict of interest that could be perceived as prejudicing the impartiality of the research reported.

## FUNDING INFORMATION

National Key R&D Program of China (2016YFC0905500, 2016YFC0905503); Science and Technology Program of Guangdong (2017B020227001); Chinese National Natural Science Foundation (81772476, 81602005, and 81872499); Outstanding Young Talents Program of Sun Yat‐sen University Cancer Center (16zxyc04); Central Basic Scientific Research Fund for Colleges‐Young Teacher Training Program of Sun Yat‐sen University (17ykpy81).

## ETHICAL APPROVAL

The study was conducted in accordance with declaration of Helsinki, and was approved by the Ethical Review Board of Sun Yat‐sen University Cancer Center. Informed written consent was obtained from each subject or each subject's guardian.

## AUTHOR CONTRIBUTIONS

Study concept and design: Zhang and Shao. Analysis and interpretation of data: Fang, Ma, Yin, Zhou, Wang, Bao, Wang, Wu, Hong, Yang, Huang, and Zhao. All authors were involved in the drafting, review, and approval of the report and the decision to submit for publication.

## Supporting information

Supplementary information

## Data Availability

The datasets used and/or analyzed during the current study are available from the corresponding author on reasonable request.
