# Supplementary material for: Combinatorial assessment of ctDNA release and mutational burden predicts anti‐PD(L)1 therapy outcome in nonsmall‐cell lung cancer
Source: Clin Transl Med. 2020 May 6;10(1):331–6. doi: 10.1002/ctm2.8 (PMC7240844; doi:10.1002/ctm2.8)
Supplement: Supplementary file 1 — Supplementary information [file CTM2-10-331-s001.pdf]

Supplementary Figure 1. Flow Diagram of the Study Cohort

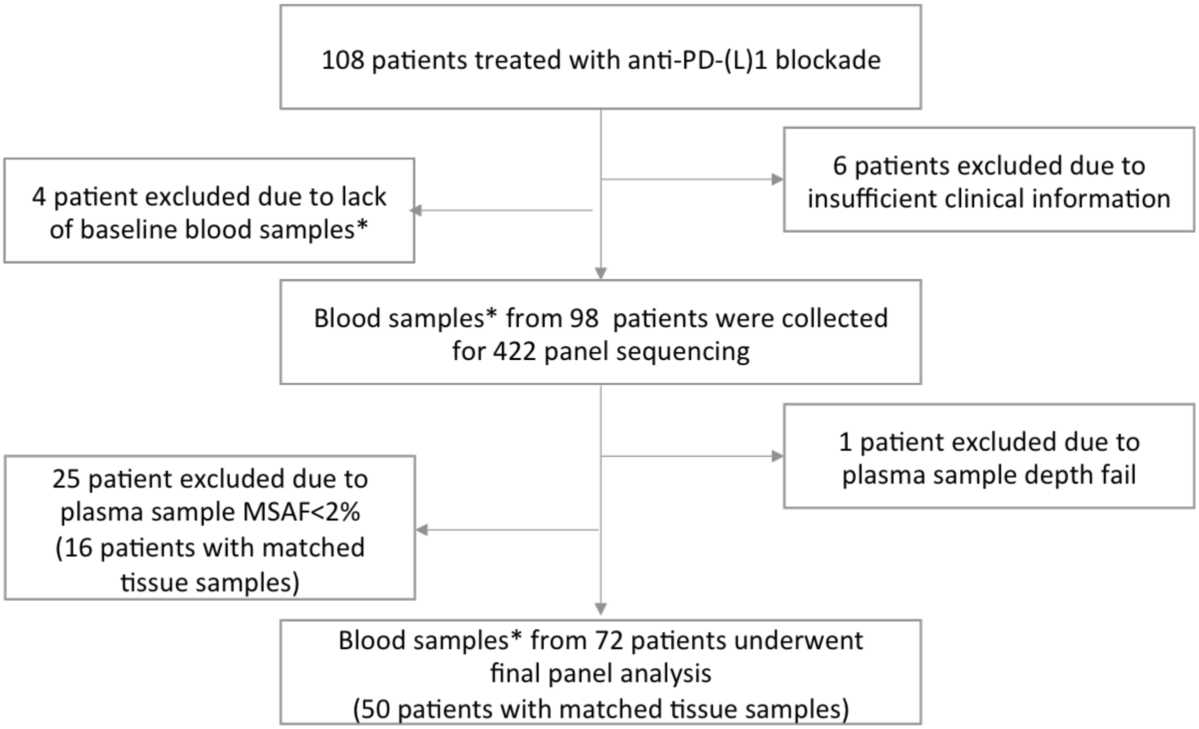

* Blood Samples: Plasma and normal control samples

Supplementary Figure 2. No Enrichment of Potential Responders by bTMB Assessment in the Entire Cohort.

Forest plot of hazard ratios (HRs) of progression-free survival (PFS) comparing patients at different bTMB cut-points. Note that at bTMB≥13, significant PFS benefit was observed in only 18% of patients with high bTMB.

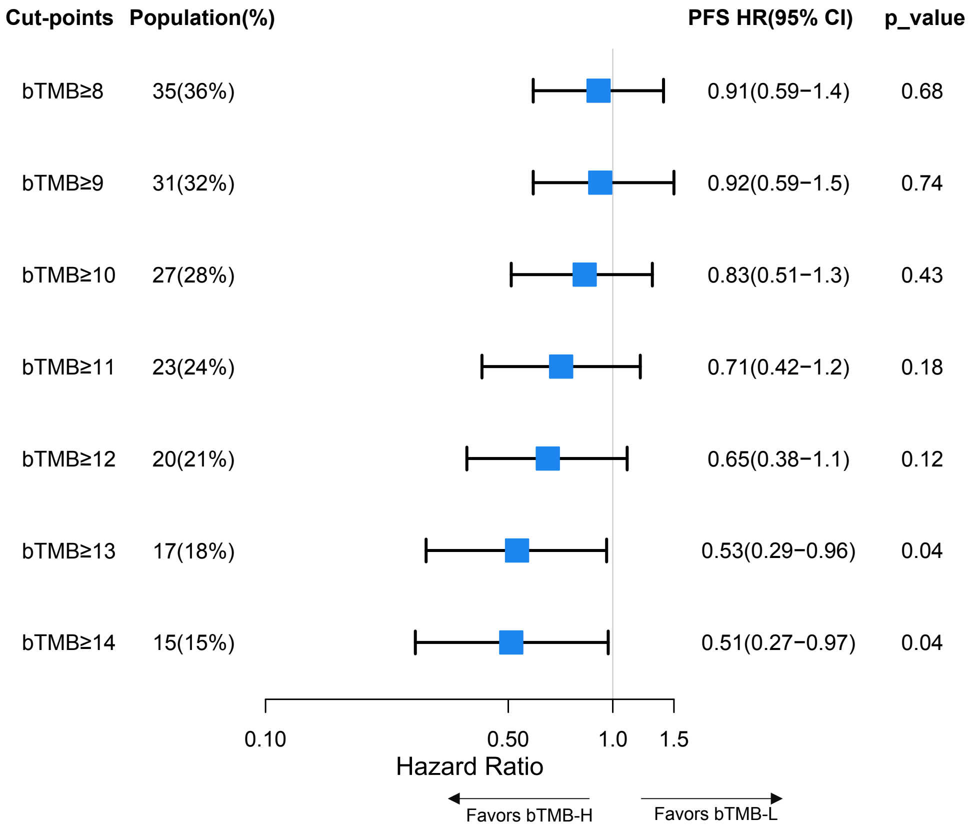

Supplementary Figure 3. Association between bTMB and ctDNA concentration or MSAF.

ctDNA concentration positively correlated with ctDNA MSAF (P=0.007), but showed no significant association with bTMB. ctDNA MSAF showed a strong correlation with bTMB (P < 0.001), which likely influenced the predictive performance of bTMB.

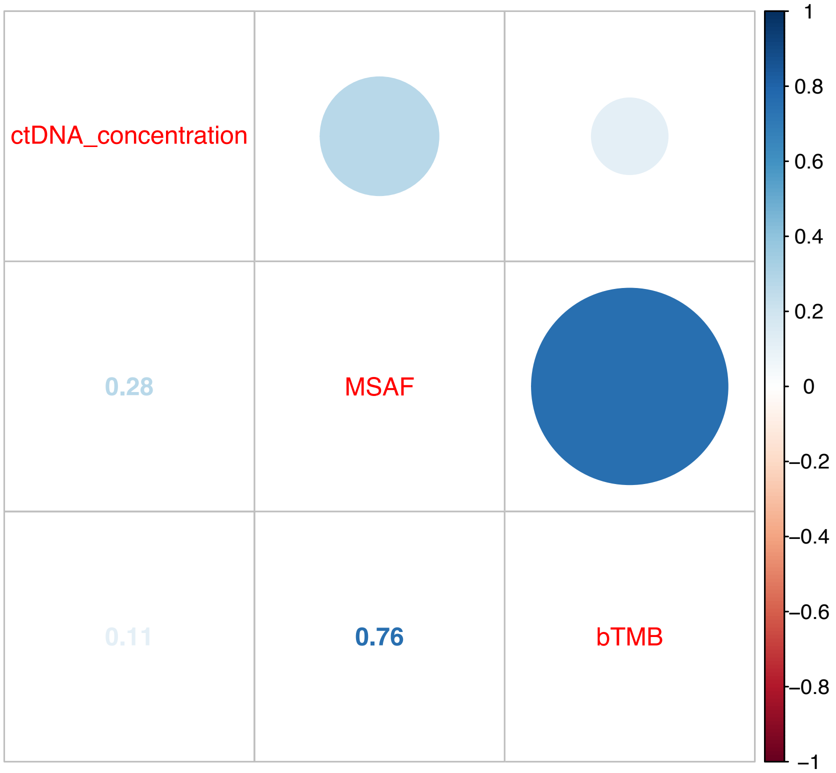

Supplementary Figure 4. ctDNA MSAF Negatively Impacts Association between bTMB and Clinical Outcome.
(A) Spearman correlation of bTMB and tTMB at varying ctDNA MSAF cut-points. Each point is indicated by the corresponding patient number (%). At ctDNA MSAF ≥ 2%, the bTMB-tTMB correlation remained above 0.7. (B) low concordance between bTMB and tTMB in the entire MSAF-unselected cohort. (C) Low correlation between bTMB and tTMB in patients with ctDNA MSAF <2%.

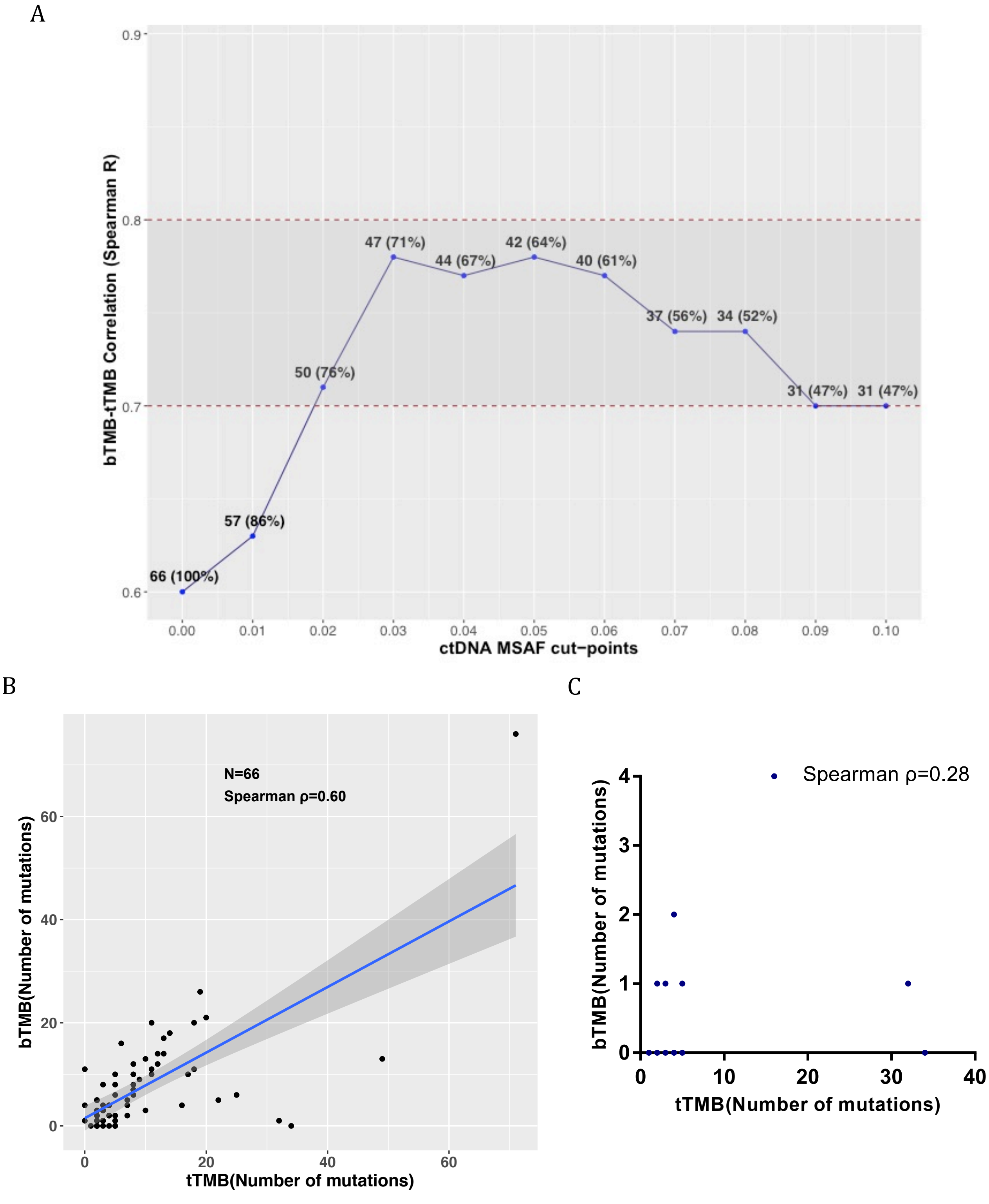

Supplementary Figure 5. Association between bTMB and Clinical Outcome in MSAF-Selected Patients.
A trend towards improved durable clinical benefit (DCB) rate in bTMB-high patients (ctDNA MSAF≥2).

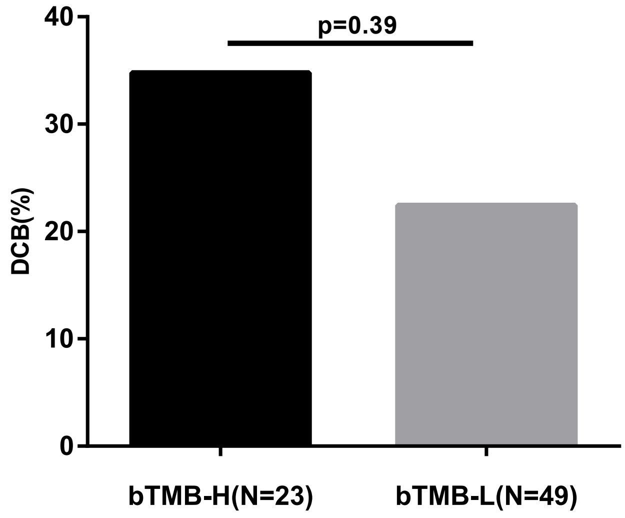

Supplementary Figure 6. Combined Predictive Value of bTMB and tTMB.
(A-B) Comparison of (A) DCB rate and (B) progression-free survival between NSCLC patients with concomitantly high TMB in blood and tissue and those with low TMB as assessed by using either sample type.

A

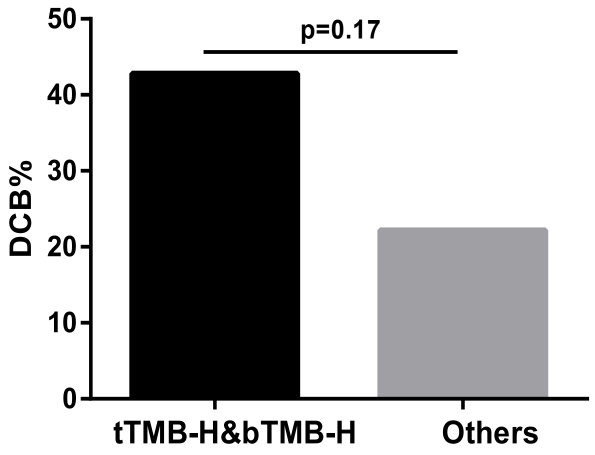

B

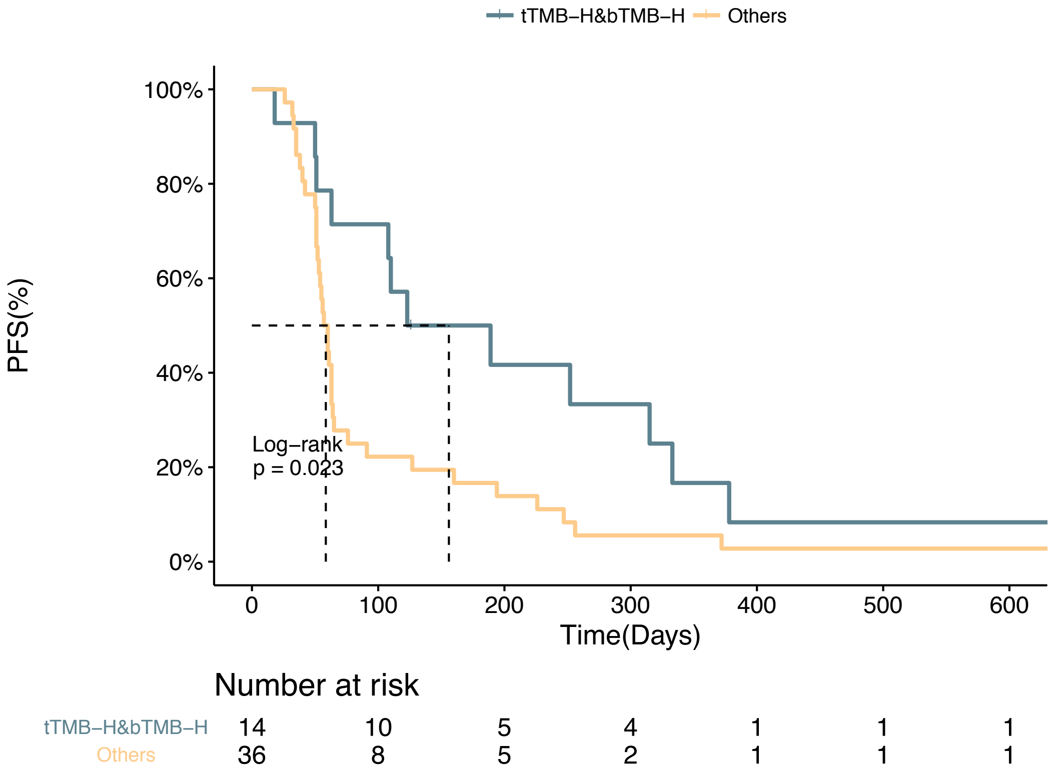

Supplementary Figure 7. Low ctDNA is Associated with Clinical Benefit from anti-PD-(L)1 therapies.

(A) Kaplan-Meier estimates of PFS in the full analysis set comparing patients with different ctDNA MSAF status. (B-C) Kaplan-Meier estimates of PFS in the (B) POPLAR and (C) OAK study cohorts. Note that MSAF-low bTMB-low patients derived similar PFS benefit as bTMB-high patients.

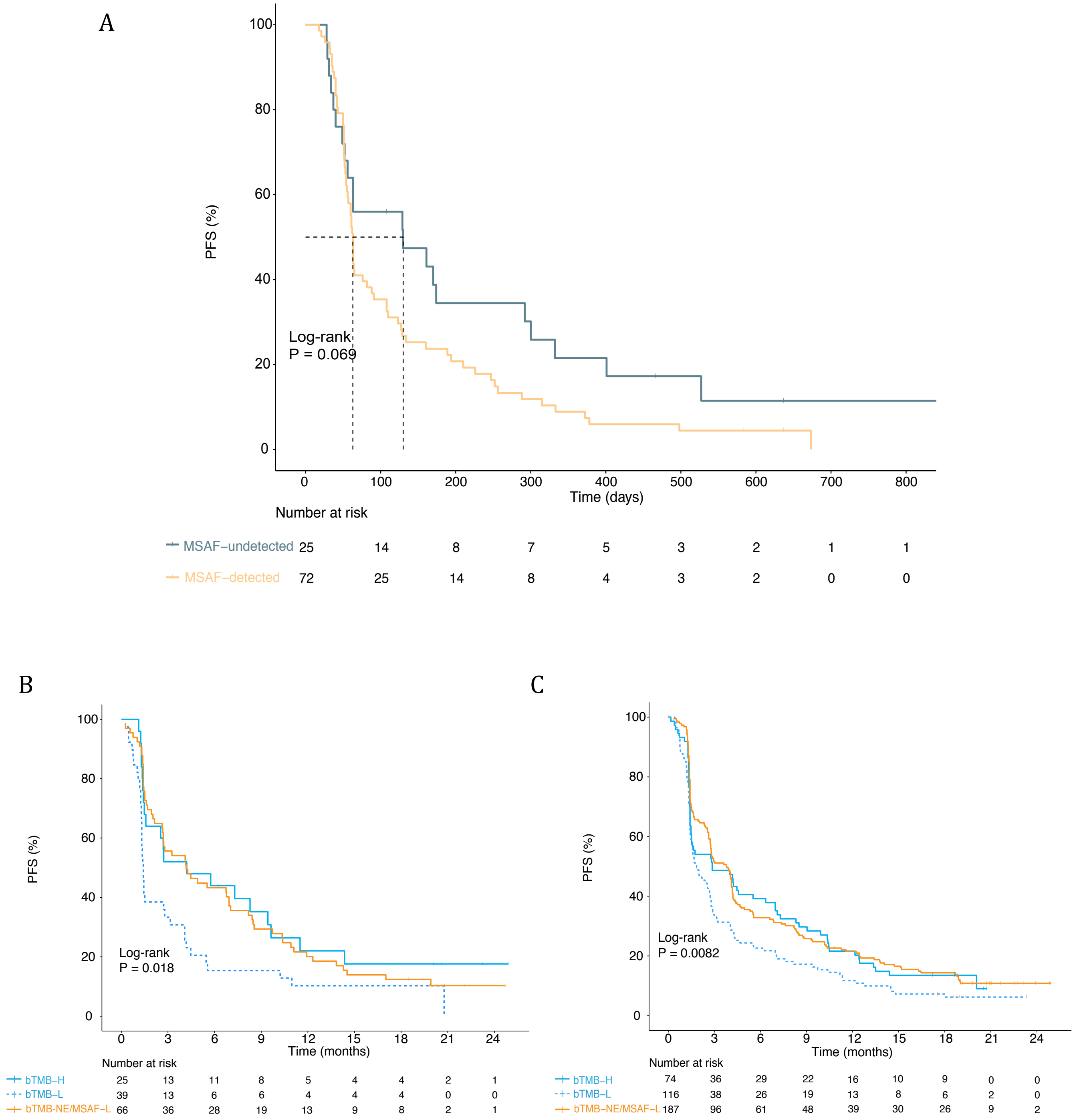

Supplementary Table 1. Baseline Clinical Characteristics of the Study Cohort.

Characteristic	All patients (N=97)
Median age (range)	54 (28-73)
**Sex-No.(%)**	
Male	61 (63%)
Female	36 (37%)
**Pathological Type-No.(%)**	
Adenocarcinoma	58 (60%)
Squamous Carcinoma	29 (30%)
Others	10 (10%)
**Stage-No.(%)**	
III	1 (1%)
IV	96 (99%)
**Immunotherapies-No.(%)**	
Anti-PD-1	93 (96%)
Anti-PD-L1	4 (4%)
**Smoking Status-No.(%)**	
Current or former smoker	45 (46%)
Never smoker	52 (54%)
